# Quarterly Retrospect

**Published:** 1861-01

**Authors:** 


### *Quarterly Retrospect.*

*January*, 1861.

"If this city, or the suburbs of the same, do afford any young gentle-"man, of the first, second, or third head, more or less, whose friends are "but lately deceased, and whose lands are but now come into his hands, "that (to be as exactly qualified as the best of our ordinary gallants "are) is affected to entertain the most gentleman-like use of tobacco; "as first, to give it the most exquisite perfume; then to know all the "delicate sweet forms for the assumption of it; as also the rare corollary "and practice of the Cuban ebolition, euripus, and whiff; which he "shall receive or take in here at London, and evaporate at Uxbridge, or "farther, if it please him. If there be any such generous spirit, that is "truly enamoured of these good faculties: may it please him, but (by a "note of his hand) to specify the place or ordinary where he uses to eat "and lie; and most sweet attendance, with tobacco and pipes of the "best sort shall be ministered: *Stet, quæso, candide Lector.*"*

We commend this advertisement of Master Shift, otherwise Signior Whiff, to the Shifts of our own day in the present stage of the so-called "Great Tobacco Controversy." Why should the æsthetics of tobacco-smoking be suffered to lie dormant? Their cultivation would afford a grand field for a man of genius. The pipe, that

"Little tube of mighty pow'r,
Charmer of an idle hour,
Object of our warm desire,
Lip of wax and eye of fire,"

must assuredly be recognised as one of the most cherished institutions of civilization; but is it not painful to reflect that, notwithstanding all the boasted superiority of the Occident, we fall infinitely below the Orient in everything that relates to the elegancy and refinement of tobacco-smoking? Compare the Eastern gentleman comfortably squatted amidst the piled-up pillows of a divan, and inhaling the aromatic fumes of the irresistible "timbac," or latakia, and a host of fragrant congeners, from a noble six-foot chibouque, or from the elegant and musical water-pipe, with the Western gentleman puffing the narcotic and irritating vapours of "shag," or "Cavendish," or "bird's-eye," from three inches of "clay," or a short-stemmed "meerschaum," blackened, it may be, with months of usage. Faugh! well may the fastidious exclaim, "The nasty, filthy, disgusting pipe!"

---

* *Every Man out of his Humour.* Act iii. sc. 3.

The ingenious and learned Democritus Junior appears to have had a vague idea that the evils of tobacco lay chiefly in the manner in which the herb was used. "Tobacco," he writes, "divine, rare, super-excellent tobacco, which goes far beyond all those panaceas, portable gold and philosopher's stones,—a sovereign remedy to all diseases. A good vomit, I confess; a virtuous herb, if it be well qualified, opportunely taken, and medicinally used; but as it is commonly abused by most men, which take it as tinkers do ale, 'tis a plague, a mischief, a violent purge of goods, lands, health—hellish, divilish, and damned tobacco, the ruin and overthrow of body and soul."*

That we take tobacco as tinkers take ale, may be a somewhat strong expression as sentiment now goes, but it is one very much to the point. If we must have tobacco-smoking,—if it is to rank among the most prominent of civilized institutions, why should not the habit be made amenable to those refinements which are to be found in wine-bibbing and cookery? History shows us that coarse feeding, gluttony, and drunkenness have declined in proportion as the arts of preparing food, and the habits of drinking wine have advanced in refinement. Let us apply the lesson to tobacco-smoking. Why should we not have a perfect art and science of smoking, and professors thereof, who, like Shift, would instruct us in what manner to give the herb its "most exquisite perfumes"? The offensiveness of the habit is a question of æsthetics, and not of ethics; of FASHION (that potent abstraction in civilized life), and not of LAW. Justice Overdo has long called aloud in the market-place,—"Stay, young man, and despise not the wisdom of these few hairs that are grown grey in care of thee. Thirst not after that frothy liquor, ale: for who knows when he openeth the stopper, what may be in the bottle? Hath not a snail, a spider, yea, a neuft been found there? Thirst not after it, youth, thirst not after it. Neither do thou lust after that tawny weed tobacco, whose complexion is like the Indian's that vends it. And who can tell, if before the gathering and making up thereof, the Alligarta hath not corrupted it; the creeping venom of which subtle serpent, as some late writers affirm, neither cutting the perilous plant, nor the drying of it, nor the lighting or burning, can any way persway or assuage? Hence it is that the lungs of the tobacconist are rotted, the liver spotted, the brains smoked like the backside of the pigwoman's booth here, and the whole body within, black as her pan you saw e'en now without. . . . . And what speak I of the diseases of the body, children of the Fair? Hark, O you sons and daughters of Smithfield! and hear what malady it

---

* *Anatomy of Melancholy.* Part ii. sect. 4; Mem. 2, sub-sect. 2.

doth the mind: it causes swearing, it causeth imagining, it causes snuffling and snarling, and now and then a hurt."*

"Are not these brave words?" exclaimed one of Justice Overdo's auditors. Brave words truly, which have long become stereotyped with those philanthropists who rank tobacco-smoking among the great evils which infest social life. But hitherto, alas! the lot that befel Justice Overdo's exercitation has befallen the well-intentioned efforts of all subsequent social reformers who have meddled hostilely with tobacco; that is to say, the efforts have been of non-effect. The reason of this is not far to seek. We can only combat error in so far as we have truth on our side. Now this simple truism has been constantly overlooked by the opponents of tobacco-smoking. They have been, and are, far too much addicted to depict the habit as one manifestly most influential in deteriorating both the physical and mental powers. Arguing chiefly from the known potent and often most noxious effects of the herb, when used medicinally, and of its essential constituents when used experimentally, it has been too readily concluded that its use by smoking cannot, under any circumstances, be had recourse to habitually without evil effects. The direct evidence in favour of this conclusion is of a most restricted kind, and is confined mainly to the influence of smoking among young persons.

This question, however, must rest upon its own merits; so also must that of the abuse of tobacco. That the use of tobacco by the young is an evil to be earnestly contended against we freely admit; that an excessive use of the herb is an evil under any circumstances is undoubted; but on the general question of the use of tobacco we would quote the opinion of Dr. Morel, the learned author of the *Traité des Dégénérescences, Physiques, Intellectuelles, et Morales, de l'Espèce Humaine, et des Causes qui produisent ces Variétés Maladives.* Dr. Morel's opinion is of peculiar value upon this question from his researches having been specially devoted to its solution—the question being one of the earliest he had to deal with in the great work to which we have referred. He writes:—

"I do not propose to attack the usage of this substance, and this for several motives. Firstly, it is far from being demonstrated that the habit of smoking or taking snuff, in proportions tolerably well understood, is in any way noxious to health. Secondly, if we examine the question with particular reference to moral hygiène, we are convinced that the most severe and efficacious legislation that could be imagined against a habit that has passed into an irresistible want, would not be without danger. The part of medicine,

---

* *Bartholomew Fair.* Act ii. sc. 6.

under these circumstances, is to indicate, on the one hand, the dangers of abuse, and, on the other, the not less great inconveniences which would arise if this substance of doubtful harm were to be replaced by another, the eminently deteriorating influence of which cannot be denied by any one. I allude to opium in these last words, and my argument for the moment may be expressed in the almost trivial truism, that of two evils it is best to choose the least." (p. 171.)

But the question of the use of tobacco does not rest, as is commonly imagined, solely upon the influence of the herb on physical health. We cannot justly conceal from ourselves the great pyschical benefits which are constantly arising from the habit of smoking. Watch the soothing effect of a pipe on the mind of the labourer in the intervals of his labour; mark how often the misery of domestic suffering, or the dread wretchedness of semi-starvation among our cottagers, are assuaged by the potent fumes. What! we hear some reader exclaim, parenthetically, a labourer waste money on tobacco when he has not wherewithal to feed himself with! Yes, dear reader, often and often have worn and wasted man *and woman* said to us, at a time when we, in our ignorance, also thought it a sinful waste to spend money upon the cherished herb, "Ah, sir! with the money that would buy but one or at the best two meals, I can eke out a week of half meals. Without the tobacco I should starve right out."

Brand quotes the following quaint old epigram;—

"All dainty meats I do defie,
Which feed men fat as swine;
He is a frugal man indeed
That on a leaf can dine.
He needs no napkin for his hands,
His fingers' ends to wipe,
That keeps his kitchen in a box,
And roast meat in a pipe."

Ask the soldier who fought in the trenches before Sebastopol, in the rain, deep mud, and slush of autumn, in the bitter cold of winter, and in the burning heat of summer, when the deep ditch glowed like an oven, what was the value of his pipe, particularly when his rations were scanty. Think you that gold would have bought it, or that he would have held up the better without it?

Beaumont sang, and our Crimean soldiers would sing with him:—

"The cold it doth heat,
Cooles them that do sweat,
And them that are fat maketh leane:
The hungry doth feed,
And, if there be need,
Spent spirits restoreth again."

Ask our sailors now, in these winter days, how they value their tobacco. Ask many of our literary, and scientific, and mercantile men, whose brains are fretted with labour, whether the pipe is not to them a magic wand. But what need to multiply instances ? The habit of smoking carries good as well as evil in its train; and if we would eliminate the evil, we can only hope to effect this by confining ourselves solely to that object, and not by confounding the evil and the good together, and indiscriminately attacking both. The weakest point of tobacco-smoking is its ethical, not sanitary aspect.

The *Daily Telegraph*, in a most able article, has dealt admirably with the latest phase of the tobacco controversy, and has set forth most clearly the chief physical evils anent smoking with which we have to contend :—

"The great Tobacco Controversy is by no means settled. Mr. Solly, Sir Benjamin Brodie, and the sages of the Anti-Tobacco Association have had their say; yet a great deal remains to be argued and explained *pro* and *con*. Those of our readers who are fond of polemics may remember that, after a well-known theologian of the seventeenth century had published his *Last Words of Mr. Baxter*, he bethought himself that he had not quite settled all matters spiritual to his own satisfaction, and so came before the public with a ponderous pamphlet, entitled, *More Last Words of Mr. Baxter*. Thus, with this precedent for an apology, we may be excused for again calling attention to the vexed question of the innocuous or baneful effects of tobacco-smoking. Our own opinions on the subject should be, by this time, pretty well known. We hold that in adults the capacity, or the want of capacity, for inhaling or emitting the fumes of tobacco must be considered as a matter of constitution and temperament. If a man's nerves or stomach cannot stand or are injured by the use of tobacco, he must either be a fatuous idiot or a constructive suicide if he ever attempts to put a pipe or a cigar between his lips. We hold that in any case smoking in adolescents should be discouraged until a young man be well grown, until his muscular system be fully developed, and his habits of life definitively formed. We hold most decidedly that the practice under any circumstances among growing boys and lads should be sternly inhibited by parents, by employers, and by those entrusted with the education of youth; and, finally, we hold that all smoking to excess is most injurious and most dangerous, and in no case more so than with persons whose pursuits are of a sedentary nature. While, therefore, we refuse to join in any crusade which has for its object the legislative repression of smoking, or the denunciation, either from pulpit or platform, of taking tobacco as a practice akin to the deadliest of the seven deadly sins, we are desirous of assuring all those who wish to abate the indubitable evils to which immoderate and premature smoking gives rise, that they have our heartiest sympathy, and that we shall continue to exert our influence to mitigate the reckless indulgence in a questionable luxury, to which so many of our youths, particularly of the middle and upper classes, are addicted.

"It is curious to note the new phase into which, within the last few months, the tobacco question has entered. Leaving Germany out of the question—which, from the Elbe to the Danube, is one vast expanse of cloud-land—the neighbouring country of France is about the last in Europe from which we should expect to hear a word alleged against tobacco. She is the favoured land of cheap and nasty cigars, of snuffy old gentlemen, of *lorettes* who puff *cigarettes*, and of short pipes *culottes* with the strongest *caporal* reeking with essential oil. The immense extent to which smoking is carried there may be judged of by the facts, that the cheapest cigar in Paris costs a halfpenny, and the cheapest cigar in London a penny; and that, while nothing is more common than to see the poorest classes in France with a cigar in their mouths, it is of exceedingly rare occurrence to see either an English labourer or soldier smoking even the humblest penny *Pickwick* in the streets by day. It is true, the private smokes in his barrack-yard, and the workman enjoys his pipe as he goes to or returns from his work; but the French artisan and the French soldier are always smoking. Abroad they puff at the coarse and rank *sou* cigar. At home they stupify themselves with the narcotic and oleaginous pipe. So apparent has this constant steeping of the faculties become to the military authorities, that the permission to indulge the habit in barracks has been in a considerable degree abrogated; but still the French warrior consumes tobacco to an extent which would amaze the most inveterate subaltern of Aldershott and the Curragh. Some very interesting statistics have, moreover, lately been published, showing, so far as figures can show, that the youths at the Imperial schools and colleges addicted to the practice are intellectually inferior to those who repudiate it;* and the Minister of Public Instruction, alarmed at the prospect of the cigar-loving pupils of the Polytechnic and the Lyceums turning out dunces, has positively prohibited the use of tobacco in the educational institutions of the State. As Paris alone contains 29,000 pupils, it may be seen to how large a population this edict applies. Public instruction in England is not so widely extended. The masters of schools such as Eton and Harrow have generally been in the habit of putting down smoking among their boys by a smart application of the birch to those detected in indulgence in this illicit gratification. In private schools, the authority of the master is usually found sufficient to prevent the evil; and no such very serious harm accrues from a few big boys occasionally stealing out on a half-holiday to enjoy a surreptitious cigar. But, although the great body of schoolboys are exempt from any of the more serious evils of excessive smoking, there are thousands of young men, either in *statu pupillari* or just entering upon life, to whom an overdue propensity to it is in the highest degree deleterious. The real victims to tobacco in England are not little boys at school, but young subalterns, students, undergraduates, clerks, assistants, and pupils to engineers and architects. These are the young sensualists whose perpetual pipe or cigar renders them pale, sickly, and haggard, stunts their growth, and turns lads who should be ruddy, lusty, and active, into decrepit striplings, whose emaciated forms and feeble gait give them equally the appearance of suffering either under confirmed ague or incipient *delirium tremens*.

"While it is expedient—and, indeed, in the highest degree necessary—to deprecate the practice by boys and growing youths, we cannot for one moment give our adhesion to any project for putting it down by the strong arm of the law. The last remnant of the 'Blue Laws' of Massachusetts was to prohibit, under penalties, all public smoking. It became eventually impossible to enforce the penalties, and we believe that the prohibition itself has by this time fallen into desuetude. Some well-meaning but mistaken people in Glasgow are striving to introduce a similar foolish and tyrannical ordinance into the second capital of Scotland, and have memorialized the municipal authorities to hamper their new police laws with a clause rendering smoking in the streets a punish-

---

* We only know these statistics through the medium of one of the daily journals; but, so far as they could be judged of under these circumstances, they are far from satisfactory. The boys who smoked, it was stated, formed the least promising material in the schools, and it was inferred that their intellectual inferiority arose from the vicious habit indulged in by them. But might it not have been that the habit was adopted only by the most stupid and idle boys? that the smoking was the index of intellectual inferiority, not the cause?—Ed. Ps. J.

able offence. The thing, clearly, cannot be done. It would be an unjustifiable interference with the liberty of the subject, and, while it might preserve a few sensitive noses from the contagion of tobacco, the domestic comfort and cleanliness of Glasgow would be in the long run the sufferers by so silly an edict; for the smokers, driven from the streets, would be eternally puffing in the interior of the houses, and every parlour, dining-room, and bedroom of the commercial metropolis of Scotland would be rendered pestiferous by the fumes of Cavendish and 'honey-dew.' The experiment of forbidding the habit has been tried in Berlin, and notably in St. Petersburg, where, after ten years' endurance, it was found to have produced the pleasurable result of making the majority of the Russian ladies confirmed smokers! 'Glasgie bodies' had better bear with the ills they have than fly to others that they know not of. An anti-smoking law in Great Britain would be as anomalous, and prove in the long run as inoperative, as a Maine liquor law; but our strong disinclination to see any undue interference with a custom which intimately affects the personal liberty of the community in no wise alters our opinion as to its noxious effects on youths, and the necessity of discouraging the practice by all legitimate means." (*Daily Telegraph*, Nov. 20, 1860.)

It is reasonable to presume that when the Glasgow anti-tobacconists proposed to legislate for tobacco-smoking as for factory chimney-smoking, they were acting under a paroxysm of social reform enthusiasm, induced by the meeting of the SOCIAL SCIENCE CONGRESS in their city. The annual gathering of this Congress has now become an established and the most notable event of the terennial quarter of the year. The last meeting, held in Glasgow, was in no wise less interesting than its predecessors. We do not propose, however, to touch upon more than one portion of the proceedings, the address, to wit, of SIR J. K. SHUTTLEWORTH, on the *Correlation of the Moral and Physical Forces*. This address contained a masterly and most philosophical outline of the great principles of social science, and these were treated with a comprehensiveness and novelty peculiar to Sir James's disquisitions. We quote an abstract of certain portions of the address:—

"Sir James began by referring to the address which he delivered last year at the meeting of the Association at Bradford, in which he drew attention to the traces of a similarity of laws in the material history of the world, and in the growth of modern civilization. The stability of nature and of society were alike shown to be consistent with perpetual change. The change was for the most part so gradual as to cause no disturbance to order. In like manner the evolution of a primeval design through long ages of development in the world of mixed moral and material forces, was manifest in the history of civilization. The co-ordination of moral and material forces in the development of civilization was no other thing than the reign of the supreme intelligence over the mixed moral and physical constitution of man. It was to this correlation of moral and physical forces in questions of social science, that he ventured to solicit attention. The marvellous conjunction of the material with the purely vital had a close analogy to that commixture of the moral and physical in man and in society the laws of which it was the peculiar function of this Association to explore. For example, pure economical science was concerned only with the accumulation and distribution of wealth. Yet such was the co-ordination of moral and physical forces in the constitution of society, that even economic logic, by severely excluding moral considerations, encountered the operation of higher laws than those regulating the accumulation of wealth—laws which demonstrate either that the sources of wealth lie deep in the moral nature of man, or that the result, wealth, may be purchased at a price ruinous to individual happiness and to the well-being of a State, because inconsistent with moral laws. Sir James proceeded to give an illustration of both of these interferences. His first illustration referred to cheap labour, in which he showed that while the cheapness of slave-labour was diminished by various moral considerations, the labour of freemen became more valuable in proportion to their willing acquiescence in the terms of the contract, to their intelligence and morality, and to their dexterity and inventive skill. The development of manufacturing industry had been as much promoted by the skill and inventive faculty of the free workman as by the master. The steam-engine was improved—almost invented—by a working instrument maker in this city—the spinning jenny was the creation of a handloom weaver—the 'water-frame' and the circular carding machine, of a barber—the 'mule,' of another handloom weaver—the 'self-acting mule,' of Richard Roberts, who had been a working mechanic—the modern railway and locomotive, of a colliery mechanic. English calico printing owed its power of competing with the French and Swiss chiefly to the discoveries of John Mercer, originally a handloom weaver, and then a self-taught chymist. These were the results of freedom, not of slavery; yet without some of these inventions probably England would have been unable to compete with Napoleon I., and Europe would, for the last fifty years, possibly, have been groaning under one vast military despotism of the dynasty which sprang from the French Revolution, to destroy feudalism and erect itself on its ruins. Here, then, a higher law, lying deep in the moral constitution of man, encountered that which was once the law, governing the relations of master and slave. Labour was not cheap in proportion as men were profligate, and willing to sell themselves, their wives, and children for the means of leading sensual lives. But labour was cheap in proportion to the intelligent exertion, the inventive skill, the enlightened co-operation, the forethought, morality, and sense of religious obligation among our freed men. Passing next to the second consideration—namely, that wealth might be purchased at too great a price, ruinous to the individual happiness or to the well-being of a State, because inconsistent with moral laws—Sir James adduced the slave trade and the opium trade as affording examples of the reaction of an immoral traffic, by introducing an element of moral and social weakness into the State. Under this head he also referred to our own laws with respect to the sale of beer and spirituous liquors, which we permitted to be used in excesses destructive to health, property, and life, brutalizing and degrading our population—excesses which might, however, be diminished by a vigorous police and a stringent administration of the law under the Licensed Victuallers' Acts, and extirpated by religious education. Universally the proposition was true, that the profits of a trade or the sum of a revenue which depended on the extent of any degrading social habit among the people, were inconsistent with their happiness and with national well-being, and also with their economic prosperity, because they destroyed the very sources of wealth—the health, energy, intelligence, and virtuous constancy of a nation. In the algebra of the science of wealth, the relations of capital and labour were considered solely as a question of supply and demand. The moral element which led to combination was kept out of sight. The science simply denounced the interference of strikes. But they were a social war, in which men were contented to subsist on a fourth part of their ordinary wages for months. They disturbed the supply of labour, and might disperse it. The influence of affable manners, attention to the comforts and well-being of workmen, perfect justice in all transactions, or still more of generosity, in attaching men to the interests of their masters, were, among many other moral forces, interfering with those which are purely economic. In like manner, experience operated so powerfully that the sons of men who destroyed newly-invented machines would now eagerly protect any mechanical improvement. The sense of social and personal right was growing so fast that the picketing of mills was at an end; personal intimidation was assuming milder forms, though it still resorted to the flagitious weapons of slander and libel. The power even of the most skilfully organized combinations of capital was met by the combinations of labour. But trades' unions could not be permitted to usurp the direction of capital. That would be as fatal to commerce as an eruption of the central fires, or the destruction of credit by the occupation of London by a foreign force. Nor could capital in a free country be permitted to dominate by prolonging the ignorance and sensuality of the workmen. Neither the domination of capital nor of the trades' unions was compatible with social freedom, or with commercial prosperity. But these forms of usurpation could only be averted by such intelligence and higher morality as might enable the parties interested to adjust with patience and good will the relations of capital and labour in their growing but ill-defined partnership. By experience and education only could the workmen be induced to leave undisturbed the control of commercial enterprises in the hands of the capitalists. By a conviction of the price which must be paid for the cordial co-operation of the workmen, and for the sense of security which was essential to trade, would the master ultimately so adjust the distribution of the profits of trade as to make the interests of the working man his own. Sir James next referred at some length to the marked influence of civil and religious freedom on the growth of our economic prosperity, and, in reply to those economists who questioned the interest we had in applying the national revenues so freely as was now done to the education of the people, he urged that it was impossible to diminish effectually pauperism and crime without employing moral as well as economical or repressive forces for their extirpation. Improved sanitary agencies did not expend their force simply in the prolongation of the mean duration of life, nor in the increase of health and vigour in the population—they reached even the moral nature of man. They were a part of civilization which rendered him less subject to debasing instincts, prepared him for the culture of his intelligence, and elevated him from brutish habits towards the transforming influence of religion. It would, however, be a fatal error to rely on sanitary agencies alone; and he proceeded to consider next the influence of education as a great moral force on national polity, and how it ought to be organized so as to be consistent with civil and religious liberty. The first question which next arose was, whether education ought, in all cases, to be regarded as a sacred function of the parent, with which none were to interfere? Individual liberty was so important an element of social freedom that we who, as a nation, preferred, before every other benefit of civilization, to be free, had the utmost jealousy of the invasion of that power of separate action which, especially when it affected liberty of thought or speech, or obedience to conscience, became with us a passion. To protect the child from parental neglect had with us, therefore, been subordinated to the personal freedom of the parent. He believed that the experience of the workmen in those trades in which the hours of labour of women and children had been limited, and the children from the age of eight to that of thirteen, had been required to be sent to school, had not only inspired the operatives with a conviction of the salutary operation of the law, but had even led the large masses of people working in them to regard, in a greater degree than heretofore, the legislative and the executive Government as a protective power, exercising a wise and beneficent vigilance over their well-being. The extension of the half-time system, or of some equivalent arrangement, to all associated labour, with such modifications as might be found indispensable to meet the peculiarities of particular employments, appeared to him the first expedient by which our great manufacturing establishments could be adequately supplied with the labour of children, consistently with that which was the most urgent social, commercial, and political want—the creation of a literate, moral, and religious working class. Apart from the proper questions which are involved in bringing all the moral forces, and among them the highest—that of religious faith—to bear on the education of our youth, the Government had wisely determined to preserve the religious constitution of our schools."

Sir James then proceeded to detail the state and requirements of education in the kingdom, and he then concluded thus:—"The sum of intellect and of religion in society were the two greatest forces by which nations might be changed. He had shown how the moral forces were correlated to all others. The moral forces, correlated with new modes of action, constantly tended to penetrate the whole structure of society. When their transforming power was witnessed, as in the history of civilization, it might be believed that this transformation, though so general in men's ephemeral lives as almost to escape observation, was still in progress surely approaching in the ages of that time which inspiration described as the era when all the kingdoms of the world should become the kingdoms of the Lord and of His Christ. Meanwhile, they might with reverence seek, as this Association did, to understand these mixed forces, and to make ourselves their intelligent senators. Sir James concluded by saying that they were not the true friends either of the State or of the people who would teach the ignorant and sensual to grasp at power. No dominion could last which was not in harmony with those primeval moral forces which operated by religion and mind, by faith and knowledge, by intelligence and virtue, on the happiness of men and the strength of States. These were the forces which must rule. They had an irresistible power. They subordinated all the physical forces to do their work. They penetrated alike the influence of race and civilization, of traditional customs and ancient institutions; they transformed the most hostile combinations; they entered the conscience of the individual man, and made him a new being. In like manner could they enter the corpse-like structure of a death-stricken nation, and make it a regenerate people. For this we had the assurance of Christ—'Ye shall know the truth, and the truth shall make you free.' Sir James sat down amid loud cheers."

Upon certain practical suggestions contained in this address of Sir James, as well as in an address of the Hon. Arthur F. Kinnaird, on "Punishment and Reformation," delivered at the same meeting, the *Times* made the following instructive comments:—

"Sir J. K. Shuttleworth says many true and important things in his Glasgow paper. He says that the art of money-making is not the only art that a nation ought to cultivate, that if we give ourselves entirely to commercial science and aim at increasing the national capital anyhow, as if that were the only thing to be thought of, we shall find ourselves coming into collision with other laws relating to national prosperity, and especially the laws of morality,—that these two sets of laws must come to a compromise and understanding with each other. This is the doctrine of 'the correlation of moral and physical forces.' The sum total of our products might gain for a time by an unprincipled and grinding system of labour which used men as brute instruments, but it would ultimately lose by this brutalization of the mass. We should never have got a Watt, an Arkwright, a Roberts, a Mercer out of our operative class, with their inventions to multiply capital a hundredfold. Our revenue might gain by an excess of consumption of spirits, but the moral deterioration of the mass would result in a loss far greater than any such gain.

"All this is very true, and it gives us great pleasure to see gentlemen like

Sir J. K. Shuttleworth and Mr. Kinnaird looking after the morals of the country. We only wish we could see less of a rising disposition to invoke Parliamentary assistance to this cause. Philanthropists, moralists, and regenerators are subject to the same temptation that all others who have plans and schemes are. They like results. They want a good tabular show at the end of every five years, and they are right in wanting it; but they go further, and are so afraid of not getting it by ordinary means that they ask Parliament to help them. Both Sir J. K. Shuttleworth's and Mr. Kinnaird's papers tend to legislative interference in morals. We note this tendency in the Social Science papers on this class of subjects, because it is a tendency against the spirit of the age, and because if this is the new Social Science policy on these subjects, it ought to be understood to be such. Sir J. K. Shuttleworth would have the crime of drinking extirpated by 'religious education, a vigorous police, and a stringent enforcement of the Licensed Victuallers Act.' Mr. Kinnaird is for placing 'crowded tenements' under penal Acts, for introducing the Forbes Mackenzie Act into England, and for a new Act of Parliament for the observance of the Sabbath. This is very intelligible. When men have hobbies, be they ever such good ones, they are anxious about them. They look on all sides of them to see what chances they have upon the ordinary ground and left to their own working. Sir J. K. Shuttleworth's hobby is education—a most creditable one. He looks about him, then, to see what chance it stands when left to nature and the voluntary principle, and he does not think much of it. What are we to do with the 'apathetic districts?' What are we to do with the manufacturer's absorption of young hands and the parent's love of the child's earnings? He goes over some Acts of Parliament that have *not* passed, which were intended to rectify these evils. This does not raise his spirits, more especially as he thinks if they *had* passed they would not have effected their object. A 'permissive' rate would have been to little purpose; only a 'compulsory rate' would have had any effect, and a compulsory rate he does not expect to get. As a step in the interim he would like to have a million more of the public money annually, and this apparently he does expect. We cannot say we do. So, what with manufacturers and what with parents, what with 'apathetic districts,' and what with 'civic authorities who do not show zeal in the cause of education,' Sir J. K. Shuttleworth is no preacher of the voluntary principle in this cause. He does not actually say that he would like an Act of Parliament to compel all parents to send their children to school and keep them there up to a certain age, but he is offended by the irregularities of the present system. Reformation is Mr. Kinnaird's hobby, and here it is the same story over again. Mr. Kinnaird is evidently afraid that we shall not reform ourselves quick enough if we are left to ourselves. He goes at once to Act of Parliament.

"We do not want, then, to throw any damper upon the zeal of our educators, reformers, and regenerators, but we must make the observation that this is rather a new policy to start. Here is an incipient school for legislative interference in morals, which our Social Science meetings appear to have formed. The school is modest as yet, and does not demand anything astounding; it would only have our lodging houses inspected to see that men and women conducted themselves with decency, and have the observance of the Sunday enforced by Act of Parliament 'on the memorial of nine-tenths of the shopkeepers of a district or parish.' But its demands will rise in time. Whether this is right or wrong, it is certainly in the teeth of everything we have been taught for a long time. The modern maxims have all been against interference of this kind, against making people good by law, and this is now the acknowledged and reigning principle. We presume it would be the orthodox doctrine even at our Social Science meetings, but when persons have a great subject to which they devote themselves this subject forms an exception.

'You *must* legislate for education, you *must* legislate for reformation, you do not hear what dreadful things are going on in lodging houses; the law must step in here; if Act of Parliament does nothing else, it must at any rate compel decency of manners.' These gentlemen cannot learn the lesson that subjects and small subjects must mainly be left to their own resources, and must be content to struggle on as they can, and get what ground they can amid the competition of interests which surrounds us. Here is the cause of education, for example—a very good cause, doubtless, a very important cause; but why is education to expect to have everything its own way, and to have the ground all cleared for it, at the cost of any other interest that comes in the way? There are hosts of serious interests that conflict with it—interests of trade, urgent necessities of parents. All these have their rights and their places just as much as education has. Education, then, must be content to get on by its own natural claims, without being helped over the ground at every difficulty by the legislature; more especially as we observe that our Social Science friends are beginning to disagree as to what education is, and what it should be. Mr. Kinnaird thinks that the committee of Privy Council, under the advice, we believe, of Sir J. K. Shuttleworth, made a great mistake in the choice of its type of education, which was a great deal too sublime for the working classes, 'unfitting them for service, and the performance of their home duties.' He thinks this 'superficial' stuff, which inflates them with conceit, should be discarded, and that 'industrial arts' should be taught instead. 'Why should not every boy know enough of tailoring, of shoemaking, of carpentering to enable him when a man and the father of a family to make his children's shoes, though he may be a painter by trade; to make the easy chair for his wife, or even to mend his own coat?' Mr. Kinnaird has evidently not much respect for the doctrine of Adam Smith, and still less for the claims of the alphabet. It is simply impossible that school children should master the hornbook and learn tailoring and shoemaking at the same time; and if they could, it would be a sad look-out for the unfortunate wearers of these amateur articles. The principle of subdivision flies confounded before Mr. Kinnaird's plan, and, expelled as obsolete and rude from English ground, it must take refuge in Otaheite. The truth is, those gentlemen who take up great subjects are dissatisfied with all results whatever. First, we are all to be made learned and scientific by education, everybody was to know everything; then it is found that this is a mistake, and another type of education comes in fashion. Whatever type of education, however, our philanthropists may adopt, we would advise them to talk a little less glibly about legislative interference either for the cause of education, or reformation, or any other. They form to themselves the image of a good nation, rather like that of a good school under the best superintendence. They picture all the lower orders being under discipline, and obeying the advice of philanthropic societies backed by acts of Parliament, all inhabiting model lodging houses, all respectable by statute. Alas! a nation is a very rough sort of material, and its virtue, when we get it, is not manufactured by act of Parliament."—(*Times*, Oct. 1, 1860.)

*Apropos* of education and of Sir J. K. Shuttleworth's "apathetic districts," the following summary and estimate of the value of the last year's doings of the *Book-hawking Union* are of great interest:—

"Lord Brougham repeated at Glasgow his own familiar saying, 'that the schoolmaster is now abroad.' Education has long had a hierarchy of its own, but there is room also for an itinerant element, more or less analogous to the preaching friars of the thirteenth century. The 'Book-hawking' system is one of the many subsidiary agencies which have lately sprung up in aid of the main movement, and seems to do a great deal of useful work in a very quiet and unambitious way. Its object is to supply cheap literature of a healthy kind to a class who would otherwise read nothing, or only the most pernicious trash. The marvel is that, instead of having been 'started by the present Bishop of Rochester, then Archdeacon Wigram, in 1851, this should not have been one of the earliest schemes of philanthropists and educationists.' The stationary and semi-barbarous state of our rural population is not much affected by National schools. Generation after generation grow up with no aspirations beyond the condition of a peasant, and no interests beyond their own village gossip. Religion alone has elevated their ideas, but religion, after all, is more concerned with the heart than with the head. It sometimes strikes one as inconceivable what a ploughboy, even if he has been taught to read and write, can find to think of—what castles he can build in the air, what mental relief he seeks from his daily drudgery. Most occupations, including those which are purely manual, contain in themselves some elements of education, but they cannot satisfy the craving, where it exists, for a knowledge of life. This made Rasselas pine in the Happy Valley, and this, we may be sure, is no strange feeling to those who are virtually 'bound to the soil.' An abundant variety of experiences is ministered to the upper and middle classes by society and locomotion, but to the poor man there is but one avenue of approach to the outer world, and this avenue is reading. Books are to him not the symbols of intellectual task-work, but the means of refreshment, and the instruments of moral emancipation. If he reads at all, he reads in the first instance for amusement, and to gratify a legitimate curiosity. His ordinary work being mechanical, a little exercise of the mind or fancy comes as a luxury. Nor is it necessary that he should himself be 'a scholar' to enjoy this treat. 'The habit of gathering round the fire of a winter's evening to hear one of the children read out' enables many to profit by the education of one.

"The rapid progress of book-hawking almost justifies the hope that it is 'one of the new threads introduced into the old pattern of agricultural life.' In nine years it has developed 'more than sixty local associations,' and is still spreading. It is organized on a system by which a central union assists without tyrannizing over the efforts of local societies, supplying information and facilitating the collection of statistics. Of these the two most important heads are the comparative demand for books in the different classes of society. 'The largest amounts of total sales in any one district for the twelve months are:—Sussex, east, 680*l.* 13*s.* 4*d.*; Dorsetshire, 400*l.*; Hants, south, 283*l.* 17*s.* 2*d.*; Derbyshire, 349*l.* 18*s.* 6*d.*; Cheshire, 364*l.* 12*s.* 7*d.*; Durham, east, 256*l.* 2*s.* 9*d.* Some of the largest average weekly sales by one hawker are—Dorsetshire, 8*l.*; Sussex, 6*l.* 15*s.*; Durham, east, 6*l.*; Hants, north, 5*l.* 5*s.* The total amount of orders received at the central depôt (8, Paternoster-row) for the six months ending March 31, is 2,800*l.*, not including the publications of the Christian Knowledge and Religious Tract Societies, which are supplied direct from the repositories of those bodies. The average sale per annum of each society is calculated at 230*l.* Multiplying this sum by the whole number of societies, it would appear that literature to the amount of 12,000*l.* a year is being circulated by this agency in England and Wales.' It further appears that 'the great bulk of the customers belong to the classes of labourers and servants, who would never think of ordering a book at a bookseller's,' and this fact is borne out by the great preponderance of low-priced books among those purchased. This is so far satisfactory, for every one must see that the quantity of books sold is of less importance than that they should 'go to the right quarter.' 12,000*l.* a year is but a drop in the ocean of bookselling transactions, but the creation of a taste for reading in a new class is worth a great deal.

"There are two or three features in the operation of this unpretending movement which deserve notice. The object of the Association is the sale, and not the gratuitous distribution of books. In these matters the difference between charity and fair trade is fundamental. The recipient of a tract knows that the donor has some object in giving it, and, though that object be his own good, his feelings slightly rebel against it, and the more so if it be written in an affected style which he knows to be a clumsy attempt to imitate the peculiarities of his own way of thinking and speaking. 'Tracts which are given away are not sure to be read,' and if read they will not have the relish of that which the labourer has selected for himself and bought with his own savings. 'The books sold by the book-hawkers are the exact measure of the reading appetite of the people.' It follows as a corollary, that the selection, however carefully made, cannot be too miscellaneous. We are glad, therefore, to hear that 'the lists are copious, and present a wide variety to the choice.' It is not that the cottagers themselves are really capable of exercising much discrimination; but the protective system by which some busy clerical board docks and expurgates English literature till a residuum is left conformable to their notions of safety cramps the action of many a book-club intended to be popular. Among the new ideas which 'the schoolmaster' has introduced is the pretty general belief that there are more lawful things in heaven and earth than would pass the censorship of a mixed committee of Puritans and Anglicans. 'Some clergymen object to all publications of the Society for Promoting Christian Knowledge; others to all those of the Religious Tract Society.' These jealousies are much less common to the religious laity, many of whom would admit that for this purpose 'no books are good but such as have life and nature in them, and are capable of taking hold of the imagination and calling up the intellect.' But the fact is that at present the religious books are most in demand. We are even told that among the colliers of Durham 'Butler's Analogy' is largely sold; while, on the other hand, we hear that 'the book-hawkers are continually asked for books which cannot be named.' This was found to be the case when bookstalls were first established at railway stations. But, 'in proportion as the book-hawker beats out of the field the old itinerant pedlar, the circulation of such books may be checked.' The sneers that are sure to be provoked by any scheme of 'world-bettering' are powerless against an attempt to supply a real and spontaneous demand. The sufficient reason for persevering in it is that it is answering its purpose without trespassing on ground already occupied. While the great educational parties are settling the plan of their crusade against ignorance, and preparing to annex what Sir J. K. Shuttleworth calls the 'apathetic parishes,' they will do wisely to disdain no means of weakening the enemy by a less regular method of warfare."—(*Times*, Oct. 3, 1860.)

The recent revivification in the columns of the *Times* of the question of labourers' cottages serves also aptly to illustrate several of Sir J. K. Shuttleworth's remarks, and is of considerable importance. It would seem to be a natural law that human efforts must flag, must fall into a humdrum, routine condition, be their object even of the best, if they are not roused from time to time into additional activity. It might have been thought that after twenty years' strenuous labours on the part of sanitary reformers, after twenty years of an almost incessant stream of books and papers, private and public, on the shortcomings of the dwellings of the working-classes, that there was little fear of the question being suffered to fall into a comparatively lifeless state in any district or town. The evil is one, however, that has been the growth of ages, and the time of its rectification will, unfortunately, not be counted by a single, or, indeed, many scores of years. Much has been done, but more remains to be done; and it is but too certain that in many localities the question has been suffered to fall into the background, and that in others it has never been entertained but under the pressure of a pestilence. It is well, then, that our chief organ of public opinion has taken up the subject once more, and we trust that as time and occasion may serve, it will again and again bring its powerful influence to bear upon the possessors of cottage property. The following article is admirably conceived:—

"When a subject of general interest is once stirred in these columns one invariable result is, a full statement of all the difficulties that surround it. No doubt, a similar result in all ages has suggested the proverb that 'in the multitude of counsellors there is wisdom'—at least caution and circumspection. It is evident that we shall soon know all that is to be avoided, and all that is impossible, in the matter of cottages for the labouring poor. Some of our correspondents give us valuable advice, and others advice the value of which consists in the fact that it is given and should not be taken. We would not advise any country gentleman anxious to improve either his people or his cottages to let ten acres on a perpetual lease to any speculative builder, and advance him 1000*l.* to build ten cottages, to pay him 30*l.* a-year. He will thereby not only part with the virtual ownership of his land and lose all control over its inhabitants, but raise a probable nuisance for ever on his property, which he may shortly have to purchase back from the builder at the cost of another 1000*l.*, if the builder is modest enough to be bought out at that sum. We would not advise a country gentleman to place his cottages absolutely under the management of his tenant farmers, though he will naturally consult their interests and comfort in letting them. We are not prepared to recommend that all the cottages in the country shall be placed under the county surveyor or a Board of Health, without whose annual certificate no cottage shall be let or inhabited. Indeed, we frankly own we are at a loss for any legislative remedy, and can only suppose that this is a matter in which public opinion is the best remedy. At all events, the bare discussion, the facts it elicits, and the contributions of experience and common sense, must do a world of good. There are many considerations that speak for themselves, and only require to be published. The clergy, the medical profession, the more enlightened farmers, and the resident gentry, are virtually officers of health. Surely it is sufficient, for example, to call their attention to the bare fact of the windows in many new cottages not being made to open, and there being absolutely no escape for the air in the room except the doorway at the head of the narrow stairs. It is a very important remark made by one of our correspondents, and applicable to every class of dwellings as well as cottages, that our improved architecture has itself done harm. An old cottage let in the wind at a thousand crevices—between the bedroom boards, at the windows, round the ill-fitting doors, and down the spacious chimney,—while the bedroom ran high up into the thatch. In the modern cottage the close and seasoned floor, the ceiling, and the well-fitted woodwork, answer the opposite purpose of shutting out the air and rendering the bedroom little more than a good sized vault.

"But, we repeat, we must consider this a case for public opinion, and gentlemen who think public opinion apt to be impertinent and intrusive, would do well to reflect whether they would prefer the annual visits of an official sur-

*b*

veyor, or perhaps an Act of Parliament out of hand, condemning every cottage on their estates, and compelling an expenditure of 10*l.* a-cottage, under official inspection. Nobody, except a very sublime Christian indeed, or a man on the look-out for a quarrel, likes to be told of his faults, but it may save him from disasters, or what he may care even more for,—public exposure. Thus it is a painful fact, that at the very moment when we are all priding ourselves on the sanitary improvement of the country, there is an unusual amount of fever and other epidemics. Something obstinately resists all our exertions, and seems to mock the boast of science that fever is a preventable evil. 'Is it?' says the fiend; and in the newly-built, slated, and whitewashed cottage, standing high and dry, amid roses and loaded apple-trees, far away from ditches, first one droops, then another, and typhus has possession. 'There *must* be something amiss,' says the youthful curate. No doubt there must be, but neither he nor any man can say what it is. In vain they sweep, and clean, and scour, and fumigate, and establish a cordon round the devoted dwelling, and use pounds of chlorine; there is another case fifty yards off, then another, and all down the road and up the lane there creeps the stealthy foe. The visitation does its work and dies out; but as it came, so it goes, shrouded in darkness, and leaves only a few weak surmises as to its cause, and the preventives. One thing only is evident,—that, while a dozen cottages were visited, the squire, the clergyman, and the farmers, with their families, notwithstanding frequent contact with the sick and dying, did not take the fever. So it is too true—'There *must* be something amiss with the cottage.' What is it? The question cannot be answered, though perhaps if any lady or gentleman, with the delicate senses of their class of life, were to try the experiment of occupying an ordinary cottage in a village almost any month of the year, they would be able to throw some light on the difficulty.

"From a pile of letters which our space will not allow us to insert, we must take one contribution to the most gloomy, and at present most incurable part of the question. There is that which is worse even than typhus, and which is a much more common result of bad and inadequate housing. When all ages and both sexes are huddled together as close as dogs in a kennel, decency becomes out of the question, and with decency the great safeguard against moral corruption. It is needless to assert what must be the results, because there the results are, and infinite scandals proclaim a deep-seated canker in the social condition of the labourer. It is nonsense to suppose, as we have heard it argued, that nature and habit are so wholly different in the case of the labourer and his growing sons and daughters, as compared with the higher classes, that the continual scenes of one common bedroom occupied by all sorts of people have no effect on the hard rural mind. What was to be expected does happen continually, and, what is worse, is thought nothing of. As to the results, we have an important testimony in the following passage from the Report of last October 15, by the Chaplain of the Gaol at Berwick-upon-Tweed:—

"'There still remains one most important improvement to be effected before we can expect domestic comfort and family ties to produce among the labouring and poorer classes their inestimable influences for good. I refer to the miserable condition of the dwellings of some of our poorer neighbours. It is almost impossible to bring up a family to virtue and piety while they are huddled together in one wretched and ill-ventilated room. Frequently, too, the head of the family finds himself so uncomfortable at home that he seeks for cleanliness and comfort in the publichouse. Some of the places inhabited by our fellow-creatures are so miserable that neither health nor decency can possibly be retained there. The only *remedy* which suggests itself for this our greatest national deficiency appears to be in *legislative powers being given to Boards of Health* to close any house or room which is found, upon proper medical and other inspection, to be unfit for human habitation. It is indeed high time that our hard-working fellow-countrymen should be *defended from the wretchedness entailed upon them by living in rooms where any one who valued a good horse would not allow him to be stabled for a week.'*

"Whether the Boards of Health would meet the case in rural villages is a point on which we may be permitted to express considerable doubt. At present what is chiefly required is to raise public opinion against the practice of stabling our human labourers worse than the animals they drive or tend. Of course, where there are many proprietors, and where an 'open parish' invites the crowd that can find neither work nor lodging elsewhere, it becomes no man's duty to build cottages, and the work will not be done. There is, however, one simple rule universally applicable. It is that everybody should see to the proper housing of the people whom he permanently employs. True, it costs money, and the money is not always at hand. We ventured the other day to set men against land, and we deprecated the idle vanity of enlarging a territory when there was nothing in the condition of the people to be proud of. By any standard that an English gentleman can be prepared to stand or fall by, it certainly is much better to spend 5000*l.* in fifty good cottages than to add a hundred acres to an estate already estimated by thousands. In most counties of England the fifty cottages will be quite as good an investment as the hundred acres, even without considering the moral results. But it is not necessary to build new cottages in every instance. Old cottages can be enlarged and repaired, and, as a correspondent observes, supplied with good drainage, for 4*l.* a-cottage, and casements that will open for another pound. A little money will go very far in the cause of humanity, good morals, and health." (*Times*, December 18th.)

The chief thing to be noticed in this thoughtful article is, that the writer most justly insists that "this is a matter in which the public opinion is the best remedy." Legislation may smooth, and, in fact has smoothed, over many difficulties, but it will not touch the core of these difficulties. The essential obstacle to be contended with is the insensitiveness of the cottage-landlord to the moral responsibility involved in the relation between himself and his tenant. In so far as this insensitiveness is due to ignorance, it is for the doctor to diminish or remove it; in so far as it is due to indifference, for the clergyman and all right-thinking men. We hope that the *Times* has struck a note, the vibrations of which will not merely be not allowed to cease, but which will be steadily increased in magnitude.

The *Times* also has recently given place to a subject, the importance of which, in respect to the physical and moral well-being of a people, cannot well be exaggerated. We shall content ourselves for the present with simply quoting the statements of the leading journal:—

"Every change of our variable climate brings some defect, moral or physical, into broad relief. A hard winter shows us houseless wretches dying in the streets, and sets us to build refuges for the destitute; a dry summer brings drought into our houses and pestilence into our cities, and sets us to mend our water-supplies and to drain our houses and our rivers; a wet summer makes us repair our roofs and drain our lands, and look to the Registrar-General's Return in districts much visited by agues. We pride ourselves upon being a practical people, and the characteristic of a practical people is never to interfere with a nuisance until it has become unbearable, and never to set seriously to work upon a remedy until the evil has risen to a *maximum.* The interjectors of difficulties always prevail with us, until public opinion has arisen to the flood-mark, when they are, of course, swept away, and the remedy is then for the most part improvised in a considerable hurry and without much knowledge, and in a manner which almost always requires to be made workable by the aid of half-a-dozen Amendment Acts.

"Judging from the correspondence which has fallen upon us since we some days since reported a meeting presided over by the Earl of Romney, there is just now great excitement among our agricultural population concerning a question of which all the rest of the nation know actually nothing. Mr. Bailey Denton tells us that these agitating landowners and farmers represent more than twenty millions of acres of land, and the facts laid before us indicate that these lands produce at present more agues than corn-ricks, that a large portion cannot be drained, and that the drainage of the remainder must render still worse the condition of those which are so nearly hopeless. The 'general reader,' however, glances at all these facts and figures with a puzzled air, and asks the next person he meets what 'arterial drainage,' and 'out-falls,' and 'river drainage' mean, and what those incomprehensible and unreadable letters in the *Times* are really about. As we believe this to be a very important national matter, and as we know that the first difficulty with the English public is to get the general nature of a new subject understood, we will attempt to diffuse a loose idea of what is about to be pressed upon the Ministry and upon Parliament.

"If the surface of this island were exactly in the form of a plum-pudding, there would be no difficulty about draining it. We should have the wartershed line where the bit of holly is stuck in, and the water would roll down equally on all sides, just as the brandy-sauce rolls down, until it finds its ultimate destination in the ocean or in the dish. But that, as every excursionist may have observed, is not precisely the case. We have mountains and hollows, we have broad rivers and little rills, we have streamlets that run northwards to fall into rivers that run southwards. We have in many places large districts so surrounded by uplands that they at some time must have been lakes, and would be now, but that their waters have forced a narrow channel through the higher lands, through which a stream now passes and drains off the bulk of the water. There are also broad plains, which are not much higher, or are even lower, than the embanked river which runs through them ; and these plains, receiving the drainage of the neighbouring uplands, cannot drain into the river at the level at which it passes them, but might, if allowed, drain into it at its lower level, a few miles further down its stream. Now, all the districts thus situated are in wet seasons, and for the most part in ordinary seasons, in the condition of a well-saturated sponge. But why does not the foolish owner drain them ? That is just what he asks to be allowed to do. How can he drain them ? Water has an inconvenient habit of insisting upon flowing down hill, and the river that passes him is, perhaps, banked up to a higher level than his lands. True, the river also must have its fall, but to get to the spot where its level is lower than the spongy land, or perhaps even to get to the river at all, he must dig a drain several hundred yards, and all that intervening land belongs to some one else. By what means is he to get over his neighbour's land ? He must not dig it *up* to his neighbour's land, and send the water to spread over his fields. Nor would it pay him to go to all this expense for his own small farm, and to drain all the district as well as his own farm, paying the cost out of his own pocket. He might just as fairly be expected to establish a private workhouse to keep the beggars from his gate. But, even if we suppose that he were content to buy the land, and to cut the drain, and to go right away to the river, he will find, when he gets there, that there is a milldam right across it, which keeps the water back to a certain fixed level, driving back the new volume of water which he has brought to the river, and spreading it again over the country. The miller has a vested right in his water power, and there is no class with higher notions of the value of their vested rights. He will have to buy the mill, or to buy the water power by endowing the mill with a supply of steam power, or by making the miller compensation reservoirs. Somehow or other he must deal with the mill.

"Now, this, we take it is, very loosely and generally sketched out, the evil which the landowners and farmers and the sanitary reformers are now banded together to reform. They want to make the courses of our main rivers, the arteries of our water circulation, free in their direct course; they want to greatly modify the obstacles which dam up the current and throw back the waters, raising their levels in the upper country, and preventing the veins of our drainage system from flowing into them. The simile is very bad anatomy, but let it pass. They want, moreover, to get access to these rivers or streams, which are our natural drains, and for this purpose they want to carry their surplus water through the intervening lands which belong to other persons, frequently infants or persons of limited estates, who have no power to sell permission except under very expensive processes. They want to do all this at some practicable expense, and there is no other way of effecting this purpose but that which is now the principle of all modern legislation,—that of giving to a large majority power to bind a small minority, with, however, power of appeal by the minority to some public authority whose duty it should be to see that the power is not abused. It seems reasonable. The only difficulty is in working it out. To bring it to a successful issue will require knowledge, industry, and tact. The millers are a very potent interest, and they do not see their advantage in the matter; it will be a matter of considerable difficulty to provide that a man shall only be rated in proportion to the benefit he derives from the works. These, and many other obstacles not noticeable at a distant view, start up as we approach the subject. The importance of the object is, however, great enough to repay us the labour of conquering these obstacles. Want of drainage is synonymous with disease. Why is the Coast of Africa so deadly? On account of the stagnant marshes by the sea. Why is the interior so deadly? On account of the oozy soil by the side of the rivers. Why can we not land in Sicily without being struck down by fever? Again, the undrained marshes. All over the world it is the same. We see its influence intensified into death where a hot sun comes to force out the malaria; we see it mitigated into agues, rheumatisms, and fevers where a cold climate discourages the exhalations. But, although it varies in degree, the principle is the same, and, if this subject deserves the attention of the Government upon economical grounds, its claims are still greater on account of its immediate relation to the health and well-being of the population." (*Times*, Dec.20.)

Before closing our retrospect, the subject of the plea of lunacy demands a brief notice at our hands. In a leading article on the execution of MULLINS, the Stepney Murderer, the *Times* made the following remarks:—

"It is satisfactory that for once the plea of insanity has not been set up on behalf of the prisoner. We have no means of knowing on what grounds the memorial of the Sheriffs of London in his favour was founded, unless it were his own sustained protestations of innocence. But this time we have heard nothing of 'maniacal impulses,' and the records of medical science have not been ransacked to prove that taking away the life of a miserly old woman, and subsequently making a 'plant' of the stolen goods on a neighbour's premises, is one of the commonest indications of mental derangement. What makes this hypothesis so peculiarly formidable is not its absurdity, but its deceptive approximation to the truth. A homicidal intent is in itself something abnormal, and is hardly consistent with a sound state of mind; but the unsoundness which it implies is widely different from that which interferes with responsibility, and may well change indignation into pity. Unfortunately, this is only one of the ambiguities by which juries are mystified and public opinion bewildered in such cases. For instance, a medical witness is put into the witness-box for the defence precisely because he is known to entertain a scientific crotchet favourable to the prisoner. The jury are not aware of this, and, looking upon him as a tolerably fair representative of his profession, listen with respect to opinions which would make the College of Physicians stare. Another chance *in favorem vitæ* is afforded by the entire publicity of all trials. No sooner is the report in the hands of the public than all the ingenious people whose lives are spent on riddles and chess problems rack their brains for a possible solution consistent with the innocence of the accused. The inventive faculty is enlisted in his behalf, and the Home Secretary is expected to entertain any reasonable suggestion, come from what quarter it may. All this is very right, and does credit to our humanity. We merely point out the fact that no one now suffers the penalty of death till every interpretation of the circumstances has been exhausted in his favour. As in warfare the art of defence sometimes outstrips that of attack, so in the present administration of justice the advantage, to say the least, is with the accused. If the operation of the law is thus charitably uncertain, it is doubly necessary that it should act sternly and inexorably where guilt is clearly proved. The State is not like a despot who can afford to condone private wrongs, and we have no right to gratify our compassion for a man like Mullins at the expense of those whose lives literally depend on the prompt expiation of crimes like his." (*Times*, Nov. 22.)

These observations would not, perhaps, have demanded specific notice, had it not been that, after the trial of YOUNGMAN, the Walworth Murderer, the *Times* had expressed observations somewhat similar in character, and equally calculated, we hold, to add to the difficulties which beset the plea of lunacy in a criminal court. Writing of Youngman, the *Times* said:—

"In this case the prisoner's story was obviously impossible. As the Judge remarked, there could be no reason why he should inflict repeated wounds upon his mother, and ultimately kill her, after he had, as he said, wrenched the knife out of her hand. The far-fetched suggestion, therefore, of homicidal monomania in the mother would not account for the facts, even if there were the least fact to rest it upon. The 100*l.* insurance settles the question of insanity as to the son. But, still, this medical craze of 'homicidal monomania' has once more been brought into a court of criminal law, and we never allow this perilous shadow to pass unchallenged. So long, indeed, as it is only the doctors who are crazy, and the juries are sound, this 'homicidal monomania' theory is but a trap for murderers. But we do not want to catch such game at the cost of breeding it. Whenever we hear this stuff talked in our courts we feel bound to remind the mass of the population what the law of insanity, as it affects the punishment of crimes, really is. The question the law asks is precisely one which, in the case of Dr. Duncan's homicidal monomaniac, must be answered in the affirmative. It is:—'Had the prisoner any right idea of the quality of the act he was doing?' The old law as laid down was, that to protect a man from criminal responsibility there must be a total deprivation of memory and understanding. In those days none but absolute idiots were held to be unaccountable for criminal acts. We have come down one step further, and there, in the interests of society, we must make a stand, if we would not make great wickedness the licence of the acts it prompts, and if we would not emancipate human passions from all control."

Both these articles indicate a singular confusion of ideas, not only on the plea, but the nature of insanity. When a writer speaks of "a homicidal intent being in itself something abnormal, and hardly consistent with a sound state of mind," one is rather alarmed at the laxity of phraseology. "*Man is born unto sin, as the sparks fly upwards*," is the dictum of sacred writers on the *normal* state of man; consequently, to use the term abnormal in reference to a purely sinful action, is not even warranted by ethics, much less pathology. The plea of insanity is essentially based upon the existence of *diseased* mind—abnormal mind, properly so called. Again, a homicidal intent or act is not looked upon, either in law or forensic medicine, as a proof of diseased mind; the character of the act, or the circumstances accompanying the intent, may lead to a suspicion of diseased mind, but the proof or probability of the existence of this has to be derived, and invariably is derived, from other sources.

Turning now more particularly to the comments on the Walworth murderer, we would remark, that nothing transpired in the course of the judicial proceedings to render it probable that Youngman was insane, but, on the contrary, everything tended to prove that he had been guilty of a diabolical series of murders. Youngman asserted that his mother had been the murderer, and that he had killed her in self-defence. The counsel for the defence based his arguments upon this statement, attempting to show the probability of previous insanity in the mother. During the progress of the case, in cross-examination, he asked Dr. Duncan, a physician, and one of the witnesses for the *prosecution*, his opinion upon the existence of homicidal mania. Dr. Duncan answered the general question by a general answer (having no relation to Youngman whatever) admitting his belief in the existence of such a form of mania, and describing it briefly in such terms as were warranted by medical experience as well as by the decisions of our own and continental courts of justice.

Setting aside, however, these understood circumstances, the very gratuitous character of the observations in the *Times*, and their exceeding inconsistency with the actual occurrences which gave rise to the casual medical opinion upon homicidal mania during Youngman's trial it is worthy of remark, that the writer entirely ignores all experience, and even the decisions of our own courts of law, as well as of continental courts, respecting homicidal mania. He converts a simple question of *fact*, which our courts are well able to deal with when raised, into a matter of *sentiment.* In this respect we regret to say other daily papers adopted a similar course. So long as the leading organs of public opinion will not, as occasion may rise, assure themselves of the actual position of medical knowledge and of legal action upon questions of insanity, so long will that unhappy vacillation of public opinion upon these questions continue. What could more contribue to foster the annoying scenes that we too frequently witness in court than opinions such as those we have just quoted? The juryman, so prompted, enters the box prepared to look upon any plea in which insanity is raised, as a mere medical prejudice, and not a question of fact, to be decided as such. The bar is without any sufficient check in the morally legitimate use of the plea; for so long as the people look upon the plea as a matter of opinion rather than fact, so long will the bar be enabled to reproduce in our courts those scenes of contending views, so detrimental to the interests of the medical profession. When the press will content itself to deal with the plea of insanity as a simple question of fact, and with the professional witnesses who have to testify for or against insanity, as with any other witness, it will exercise an immense and most beneficial influence, in guiding public opinion aright in this matter, and in restraining those unhappy exhibitions which, neither to the interest of the law or of medicine, have too frequently disgraced our courts.

